# Census Bulletin No. 10

**Published:** 1890-11

**Authors:** 


CENSUS BULLETIN NO. 10

Contains the report of the total production of quicksilver; from which it appears that the total production in the United States for ten years was 407,675 flasks, valued at $13,480,500.

CENSUS BULLETIN NO. 11

